# The LINC00152/miR-138 Axis Facilitates Gastric Cancer Progression by Mediating SIRT2

**DOI:** 10.1155/2021/1173869

**Published:** 2021-10-16

**Authors:** Jinchun Wang, Jie Wu, Lei Wang, Xuewen Min, Zhujing Chen

**Affiliations:** ^1^Department of Pharmacy, Jiangsu Health Vocational College, Nanjing, Jiangsu 211800, China; ^2^Department of Outpatient, Jurong People's Hospital, Jurong, Zhenjiang, Jiangsu 212400, China

## Abstract

Gastric cancer (GC) is the most common gastrointestinal cancer and the main cause of tumor-related death. Exploring markers for early diagnosis and new therapeutic targets is always on the way. In the last 10 years, long noncoding RNAs (lncRNAs) have been widely proved to be involved in the progress of many tumors and are regarded as potential targets for tumor therapy. We found that LINC00152, a newly identified lncRNA, was significantly upregulated in GC tissues and affected clinicopathological characteristics in GC patients. Furthermore, we observed that LINC00152 knockdown can significantly reduce cell proliferation and promote apoptosis in human gastric cancer cells. Further bioinformatic analysis indicated that LINC00152 competitively bound with miR-138 and regulated the expression of miR-138. Moreover, SIRT2 was further proved to be a downstream target of miR-138. Overall, this study elucidates the molecular mechanism of LINC00152 underlying the malignant phenotype of GC cells by mediating miR-138/SIRT2 axis, which provides a new understanding of the role and molecular mechanism of lncRNA in GC and also provides a new way for the treatment of gastric cancer.

## 1. Introduction

Gastric cancer (GC), like lung cancer and liver cancer, is the most common malignant tumor. In recent years, the number of confirmed cases has increased significantly [1]. Although the 5-year survival rate of gastric cancer patients was improved by surgery and radiotherapy, due to the fact that many patients are in the middle and the late stage when they are diagnosed definitely and have distant metastasis, the treatment effect is not satisfied [2]. Consistent with the molecular mechanisms of other types of cancer, the development of GC also involves many biological changes, including gene expression changes, methylation modification, protein posttranslational modification, and noncoding RNA modification [3–5]. Therefore, exploring and clarifying the potential molecular mechanism of gastric cancer development will provide theoretical and experimental basis for the treatment of gastric cancer at the source.

LncRNAs are a class of long-chain RNAs without protein coding capacity [6]. Since lncRNAs are widely involved in the transcriptional regulation of genes and play an important role in posttranscriptional regulation and epigenetic mechanisms, they have exerted a new role in tumorigenesis [7]. LINC00152, originated from chromosome 2p 11.2, was initially detected in hepatocarcinogenesis and proved to have potential function [8]. LINC00152 may mediate the occurrence and malignant progression of GC [9]. LINC00152 is involved in the regulation of cell cycle activity, cell migration/invasion, epithelial-mesenchymal transition (EMT), and apoptosis of GC cells [10]. A previous study has suggested that LINC00152 facilitates the cell proliferation in GC via reducing miR-193a-3p expression and elevating MCL1 level [11]. Moreover, LINC00152 was confirmed to enhance gallbladder cancer metastasis and EMT through HIF-1a/miR-138 axis [12]. In our study, we found that LINC00152 affected clinicopathological characteristics in GC patients. Subsequently, we observed that LINC00152 inhibition attenuated proliferation and increased apoptosis in GC cells. Further bioinformatic analysis and mechanism experiments indicated that LINC00152 acted as a sponge of miR-138 and then regulated the expression of downstream SIRT2 through miR-138. Therefore, clarifying the role of LINC00152 in GC through miR-138/SIRT2 axis will provide a new perspective for the diagnosis and treatment of GC.

## 2. Materials and Methods

### 2.1. GC Specimens and Cell Culture

A total of 96 GC tissue samples and 96 adjacent noncancerous tissue samples were obtained from GC patients from January 2018 to May 2020 in Jurong People's Hospital. Our study was approved by the ethics committee of Jurong People's Hospital. The clinicopathological characteristics were recorded and are presented in Table 1. All patients with GC agreed and signed the informed consent form.

Human GC cell lines (AGS, SGC7901, HGC27, and MKN45) and normal gastric epithelial cells of GES-1 and HEK293 were purchased from the ATCC (Manassas, VA, USA). SGC7901 cells were grown in RPMI 1640, and AGS cells were grown in Ham's F12K medium. HGC27, MKN45, 293 T, and GES-1 cells were cultured in DMEM with 10% FBS and 1% penicillin/streptomycin.

### 2.2. Total RNA Extraction and Quantitative RT-PCR Assay

Total RNA isolated from tissue and cell samples using Trizol reagent (Invitrogen, Life Technologies, USA) were reverse-transcribed to cDNAs using the TaKaRa RT reagent kit (TaKaRa, Shiga, Japan). The LINC00152 and SIRT2 expression was analyzed using SYBR-PCR detection system (Applied Biosystems, USA). The PCR primers used are provided in Table 2.

### 2.3. Cell Transfection

LINC00152 siRNAs and SIRT2 siRNA were obtained from GenePharma (Shanghai, China). miR-138 mimics, mimic NC, miR-138 inhibitor, and inhibitor NC were purchased from Ambion (Austin, TX, USA). Expression plasmids encoding wild-type SIRT2 (pcDNA-SIRT2) and control vectors were provided by GenePharma (Shanghai, China). GC cells were transfected with LINC00152 siRNAs, miR-138 mimics/inhibitor, or SIRT2 plasmids using Lipofectamine 2000 (Invitrogen, USA).

### 2.4. Cell Viability

A total of 5000 cells were seeded in 96-well plates and incubated at 37°C with 5% CO_2_ for 24 h. Then, 10 *μ*l of CCK-8 solution (CCK-8, Dojindo, Tokyo, Japan) was added in each well and the cells were then incubated at 37°C for 2 h. Finally, the absorbance was detected at 450 nm.

### 2.5. Transwell Migration Assays

After 48 h of transfection, the collected cells were cultured without serum and cultured in FBS RPMI 1640 cell culture medium for 24 hours. The collected cells were suspended in RPMI 1640 cell culture medium without FBS, and then, the cell concentration was adjusted to 10^5^ cells/ml. Serum-free cell suspension (300 *μ*L) was added to the upper chamber, and the RPMI 1640 medium (500 *μ*L) containing 10% serum was added to the lower chamber, and the cells were cultured in 37°C and 5% CO_2_ incubator for 48 h. After culture, 0.1% crystal violet was used for staining for 10 minutes, and then migrating cells were imaged under a microscope. The absorbance of each well at the wavelength of 570 nm was measured.

### 2.6. Flow Cytometric Analysis

The cells of different treatment groups were washed twice with precooled PBS, and then 500 UL of trypsin was added to each well to digest the adherent cells for 1 minute. After centrifugation (1000 rpm, 3 min), the cells were washed with PBS 3 times. Then, the EP tube was moved in and Annexin-V and propidium iodide (PI) (BD, USA) were added to each tube based on the operation instructions. Finally, we use Summit software (FlowJo, USA) to analyze the data.

### 2.7. Luciferase Assays

Luciferase reporter plasmids used in our study were obtained from Genome Tech Co., Ltd. (Shanghai, China). The cells in different groups were lysed using passive lysis buffer (Promega Corporation, Madison, WI). The luciferase activity was confirmed by a Luciferase Dual Assay Kit (Promega) and was normalized to the Renilla luciferase in the control group.

### 2.8. Western Blot

The cells in different groups were washed with PBS 3 times, and then RIPA lysate (containing 0.1% protease inhibitor, 1% phosphatase inhibitor, and 0.5% PMSF) was added for 30 minutes. After protein extraction, the concentration was detected and separated by 10% SDS-PAGE gel electrophoresis (80 V, 30 min; 120 V, 45 min). After 2 hours of membrane transfer and 5% BSA blocking, the membrane was incubated with primary antibodies SIRT2 (1 : 1000) and GAPDH (1 : 5000) at 4°C overnight. The second antibody was incubated for 2 hours. Finally, the enhanced chemiluminescence solution (Millipore) was used for exposure and the gray value was counted.

### 2.9. Statistical Analyses

All data were analyzed with GraphPad 8.0. Normal distribution clustering was used to establish the correlation of miR-138 with LINC00152 and SIRT2 in GC tissue samples. Spearman correlation analysis was used to assess the correlation of miR-138 with LINC00152 and SIRT2 in GC tissue samples. The statistical analysis was performed by the nonpaired *t*-test and presented as mean ± SEM. ^*∗*^*P* < 0.05 and ^*∗∗*^*P* < 0.01 were considered statistically significant.

## 3. Results

### 3.1. LINC00152 Expression Is Upregulated in Gastric Cancer Tissues and Cells

**Figure 1:**
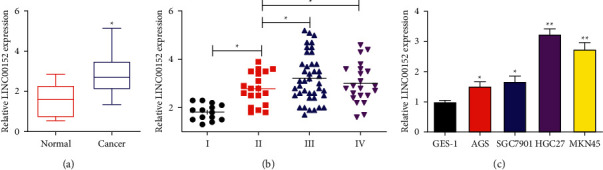
LINC00152 is significantly upregulated in GC tissues and cells. (a) Level of LINC00152 in GC tissues (*n* = 96) and adjacent nontumor tissues (*n* = 96). (b) LINC00152 expression in different GC clinical stages. (c) LINC00152 expression in control cell line and different GC cell lines (^*∗*^*P* < 0.05 and ^*∗∗*^*P* < 0.01).

We first used qRT-PCR analysis to examine the level of LINC00152 in GC patients. As shown in Figure 1(a), LINC00152 expression was significantly elevated in GC tissues compared to that in adjacent nontumor tissues. Higher LINC00152 level was determined in advanced clinical stages (III and IV) than in early stages (I and II) (Figure 1(b)). Subsequently, the LINC00152 level in GC cells was examined; compared with the GES-1 cells, LINC00152 was significantly upregulated in GC cells (AGS, SGC7901, HGC27, and MKN45) (Figure 1(c)). Of note, it was verified that HGC27 and MKN45 cells exhibited the highest expression of LINC00152.

### 3.2. Knockdown of LINC00152 Attenuates Cell Proliferation and Facilitated Cell Apoptosis *In Vitro*

**Figure 2:**
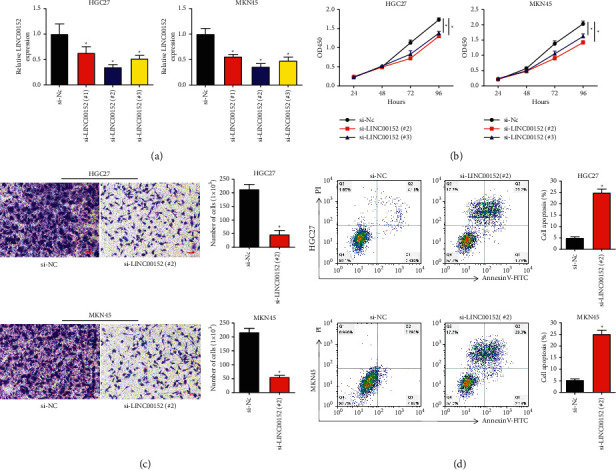
Knockdown of LINC00152 in HGC27 and MKN45 cells inhibits GC cell proliferation and promotes apoptosis. (a) Three specific LINC00152 siRNAs were transfected into HGC27 and MKN45 cells, and the LINC00152 level was examined by qRT-PCR. (b) The cell proliferation was determined when HGC27 and MKN45 cells were transfected with si-LINC00152 (#2) or (#3). (c) Migration assay analysis was performed under si-LINC00152 (#2) transfection in HGC27 and MKN45 cells (scale bar: 100 *μ*m). (d) Cell apoptosis was detected after si-LINC00152 (#2) transfection in HGC27 and MKN45 cells (^*∗*^*P* < 0.05).

To evaluate the possible effects of LINC00152 on GC cells, we silenced the LINC00152 in HGC27 and MKN45 cells using three siRNAs. qRT-PCR assay was then conducted to confirm the successful knockdown of LINC00152 in GC cells, and we found that the knockdown efficiency of si-LINC00152 (#2/3) was better than si-LINC00152 (#1) (Figure 2(a)). Therefore, si-LINC00152 (#2) and (or) si-LINC00152 (#3) were selected in the following loss-of-function assays. We clarified that si-LINC00152 (#2) and si-LINC00152 (#3) significantly inhibited cell proliferation in HGC27 and MKN45 cells using CCK-8 assay (Figure 2(b)). The migration assay results revealed that migration was significantly suppressed in si-LINC00152 (#2) cells than in si-NC transfected cells (Figure 2(c)). In addition, the flow cytometry results showed the elevated apoptosis in GC cells transfected with si-LINC00152 (#2) than in the control group (Figure 2(d)).

### 3.3. miR-138 Is Inversely Related to LINC00152 in GC

**Figure 3:**
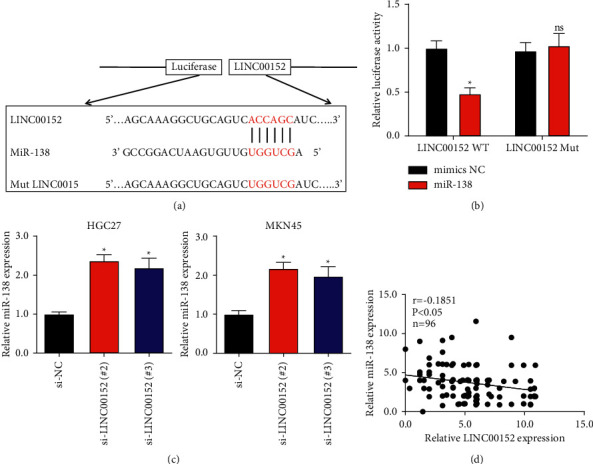
miR-138 is a target of LINC00152. (a) The putative binding site between miR-138 and LINC00152. (b) Investigation of the binding ability between miR-138 and LINC00152. (c) The miR-138 expression in HGC27 and MKN45 cells transfected with si-LINC00152 (#2) or (#3). (d) Relationship of miR-138 and LINC00152 in GC tissues ^*∗*^*P* < 0.05.

Cai et al. found that LINC00152 could exert as a competing endogenous lncRNA for miR-138 in gallbladder cancer [12]. We also discovered that miR-138 potentially bound to LINC00152 using the public website (https://www.ncbi.nlm.nih.gov/). Therefore, we deduced that miR-138 is a potential downstream target gene of LINC00152 in GC. The putative competing binding site between LINC00152 and miR-138 is as shown in Figure 3(a). Next, we used wild-type LINC00152 luciferase plasmids (including WT or MUT binding site) to determine the relationship between LINC00152 and miR-138. As shown in Figure 3(b), miR-138 could reduce wild-type LINC00152 luciferase activity, whereas it did not affect mutant activity, indicating that miR-138 upregulation promoted its binding to LINC00152 in GC cells. Moreover, qRT-PCR assay suggested that si-LINC00152 (#2) and (#3) significantly upregulated the level of miR-138 in HGC27 and MKN45 cells (Figure 3(c)), which suggested that LINC00152 could negatively regulate miR-138 expression in GC cells. Besides, in tissue samples from clinical GC patients, the level of miR-138 was negatively correlated with LINC00152 level (Figure 3(d)).

### 3.4. SIRT2 Is a Direct miR-138 Target

**Figure 4:**
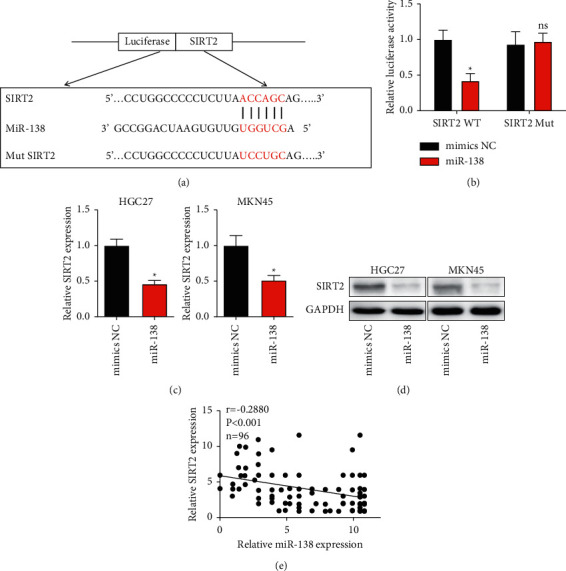
SIRT2 as a target of miR-138. (a) The putative binding site between miR-138 and SIRT2 (http://www.mircode.org/index.php). (b) Investigation of the binding ability between miR-138 and SIRT2. (c) The SIRT2 level in HGC27 and MKN45 cells transfected with miR-138 mimics. (d) The protein level of SIRT2 in HGC27 and MKN45 cells transfected with miR-138 mimics by western blot analysis. (e) Relationship between miR-138 and SIRT2 in GC tissues (^*∗*^*P* < 0.05).

Next, we aimed at identifying the main target genes of miR-138, which were predicted by using the miRcode website (http://www.mircode.org/index.php). SIRT2 was predicted to be a potential downstream target of miR-138, which has been confirmed to mediate the GC progression. We identified the potential binding site within 3′UTR between miR-138 and SIRT2 (Figure 4(a)). miR-138 was able to impair the luciferase activity of SIRT2 (WT group), while had no effect on the MUT group of the miR-138 binding site (Figure 4(b)). In addition, we further confirmed that ectopic miR-138 mimic could markedly reduce the SIRT2 level *in vitro* using qRT-PCR and western blot (Figures 4(c) and 4(d)). Besides, in tissue samples from clinical GC patients, we determined that the level of miR-138 was negatively correlated with SIRT2 level (Figure 4(e)).

### 3.5. Knockdown of SIRT2 Decreases Proliferation and Enhances Apoptosis in GC Cells

**Figure 5:**
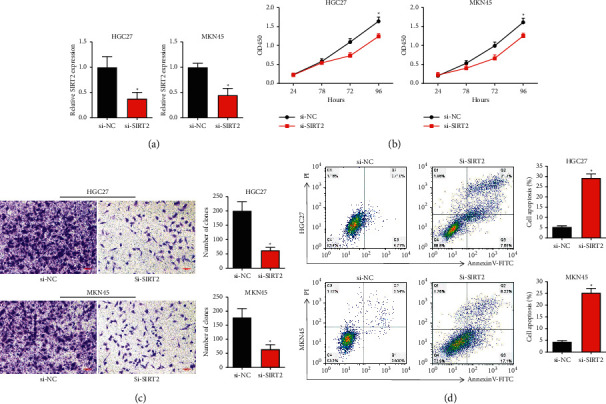
Knockdown of SIRT2 inhibits GC cell proliferation while promoting apoptosis. (a) SIRT2 siRNA was transfected into HGC27 and MKN45 cells, and the relative level of SIRT2 was examined by qRT-PCR. (b) The cell proliferation was examined after transfection of si-SIRT2. (c) Migration assay was performed under si-SIRT2 transfection in HGC27 and MKN45 cells (scale bar: 100 *μ*m). (d) Cell apoptosis was detected after si-SIRT2 transfection in GC cells (^*∗*^*P* < 0.05).

To evaluate the possible effects of SIRT2 on GC cell proliferation and apoptosis, we silenced the SIRT2 in HGC27 and MKN45 cells by a specific siRNA (si-SIRT2). qRT-PCR results confirmed the successful knockdown of SIRT2 in GC cells (Figure 5(a)). In addition, we demonstrated that si-SIRT2 significantly decreased HGC27 and MKN45 cell proliferation using CCK-8 assay (Figure 5(b)). The migration assay results revealed that migration was significantly repressed in si-SIRT2 transfected cells than in si-NC transfected cells (Figure 5(c)). Finally, the flow cytometry results showed the elevated apoptosis in si-SIRT2 transfected cells than in si-NC transfected cells (Figure 5(d)).

### 3.6. LINC00152/miR-138/SIRT2 Axis Exerted Tumor-Promoting Function in GC by Inducing Cell Growth

Both miR-138 and SIRT2 could regulate cell growth and apoptosis in GC. Moreover, we speculated that the LINC00152/miR-138/SIRT2 axis exerts a key role in the GC progression.

**Figure 6:**
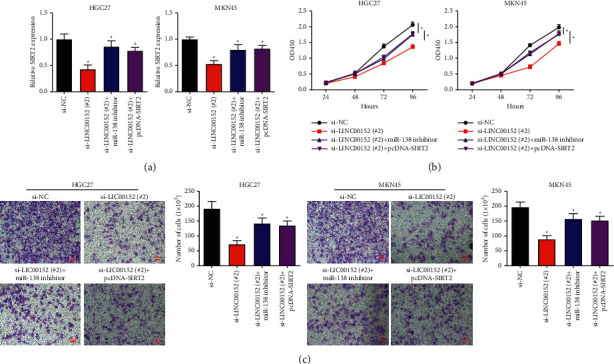
LINC00152 exerted tumor-promoting function through facilitating cell growth by sponging miR-138/SIRT2 axis in GC. (a) The relative expression level of SIRT2 in HGC27 and MKN45 cells transfected with si-LINC00152 (#2), si-LINC00152 (#2) + miR-138 inhibitor, or si-LINC00152 (#2) + pcDNA-SIRT2 was examined by qRT-PCR. (b) Cell proliferation was detected after transfection of si-LINC00152 (#2), si-LINC00152 (#2) + miR-138 inhibitor, or si-LINC00152 (#2) + pcDNA-SIRT2. (c) Migration assay was performed under si-LINC00152 (#2), si-LINC00152 (#2) + miR-138 inhibitor, or si-LINC00152 (#2) + pcDNA-SIRT2 transfection in HGC27 and MKN45 cells (scale bar: 100 *μ*m) (^*∗*^*P* < 0.05).

To confirm the regulatory effect of LINC00152/miR-138/SIRT2 axis on GC cell growth, we, respectively, transfected si-LINC00152 (#2) with or without miR-138 inhibitor and si-LINC00152 (#2) + pcDNA-SIRT2 into HGC27 and MKN45 cells. qRT-PCR results determined that si-LINC00152 (#2) significantly reduced the SIRT2 expression, and this effect was reversed by miR-138 inhibition or SIRT2 overexpression (Figure 6(a)). Moreover, the HGC27 and MKN45 cell proliferation was decreased when transfected with si-LINC00152 (#2), while the elevated proliferation was rescued after miR-138 inhibition or SIRT2 overexpression (Figures 6(b) and 6(c)).

### 3.7. LINC00152/miR-138/SIRT2 Axis Exerted Tumor-Promoting Function in GC by Suppressing Cell Apoptosis

**Figure 7:**
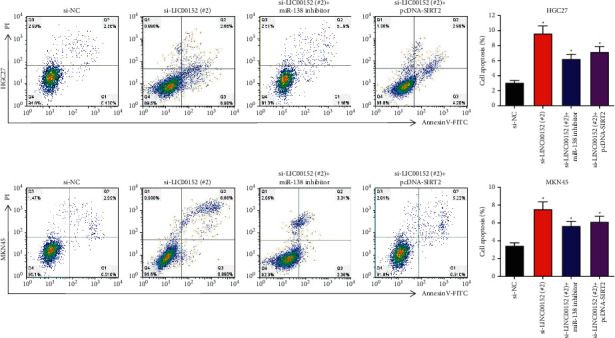
LINC00152 exerted tumor-promoting function through repressing cell apoptosis by sponging miR-138/SIRT2 axis in GC.

Subsequently, flow cytometry analysis was performed. We found that miR-138 inhibition or SIRT2 overexpression significantly reversed the promotion of si-LINC00152 (#2) on cell apoptosis in HGC27 and MKN45 cells (Figure 7(a)).

Cell apoptosis rate was examined under si-LINC00152 (#2), si-LINC00152 (#2) + miR-138 inhibitor, or si-LINC00152 (#2) + pcDNA-SIRT2 transfection in HGC27 and MKN45 cells (^*∗*^*P* < 0.05).

## 4. Discussion

LINC00152 plays a key regulatory role in GC development [10]; for example, LINC00152 could promote GC cell cycle progression and promote tumor growth [13, 14]. Moreover, LINC00152 mediates cell cycle activity, EMT, migration/invasion, and apoptosis [15]. We clarified in our study that the LINC00052 expression was significantly elevated in GC tissues. LINC00152 expression was positively related to TNM stages. Functionally, LINC00152 knockdown could attenuate GC cell proliferation while promoting GC apoptosis, suggesting that LINC00152 may play a vital role in gastric cancer progression.

Competing endogenous RNA (ceRNA) is one of the most common and main mechanisms of noncoding RNA [16] and is also suitable for interpreting the reciprocal repression between lncRNAs and miRNAs [16]. For instance, lncRNA XIST promotes GC carcinogenesis and metastasis, by sponging miR-101 [17]. Moreover, lncRNA SNHG5 regulates GC cell growth and metastasis via acting as a ceRNA for miR-32 [18]. LINC00152 also has a “sponge” role for miRNAs. For example, LINC00152 is certified as a ceRNA of miR-193a-3p and promotes GC cell proliferation [11]. We also found that miR-138 level was significantly increased under LINC00152 knockdown and there was a direct binding relationship between them. The expression level of miR-138 was negatively correlated with the expression level of LINC00152 in GC tissues, which provided further supporting evidence for ceRNA regulatory network.

miR-138 plays a crucial antitumor role in various cancers. In previous studies, miR-138-5p is revealed to be involved in regulating autophagy in pancreatic cancer [19], acting as a tumor suppressor gene on ovarian cancer cell proliferation, invasion, or migration [20], and is associated with clinical stage, metastasis of overall survival [21]. Herein, we presented evidence that miR-138 simultaneously targets protein coding genes and is competitively bound by LINC00152.

Seven SIRT homologues have been identified as SIRT1-7 in mammals [22]. Among them, SIRT2 mediates cell cycle, autophagy, necrosis, and apoptosis [23, 24]. However, the role of SIRT2 in tumorigenesis remains controversial. Li et al. found that SIRT2 overexpression attenuates non-small cell lung cancer progression [25]. Similarly, Li et al. reported that SIRT2 overexpression reduced tumor cell proliferation [26]. The above results suggest that SIRT2 exerts as a negative regulatory gene. By contrast, some studies also reported that SIRT2 overexpression enhanced tumor progression including cervical cancer, glioma, and NSCLC [27–29]. In our study, we demonstrated that the downregulation of SIRT2 significantly inhibited cell proliferation and migration in HGC27 and MKN45 cells. The flow cytometry results showed that SIRT2 downregulation promoted HGC27 and MKN45 cell apoptosis. Our results showed a potential role of SIRT2 as a proto-oncogene in GC. Also, we observed that LINC00152 modulated SIRT2 by serving as a ceRNA for miR-138.

In conclusion, our study expounded that LINC00152 expression is upregulated in GC and positively correlated with GC progression. LINC00152 knockdown inhibits the malignant phenotype of GC cells. In terms of mechanism, LINC00152 knockdown plays an anticancer role in GC by targeting miR-138/SIRT2 axis, which provides a novel target for GC treatment. Additionally, *in vitro* results will be further confirmed by xenograft tumor experiments in our future study, which can further clarify the translational significance.

## Figures and Tables

**Table 1:** Clinicopathological characteristics of GC patients.

Characteristics	No. of cases
Gender	
Male	64
Female	32
Age	
≤60	58
>60	38
Tumor size	
≤4 cm	42
>4 cm	54
Tumor stage	
I	14
II	19
III	41
IV	22

**Table 2:** Primer sequences.

Primers	Primer sequences
LINC00152	F: 5′-AAAATCACGACTCAGCCCCC-3′; R: 5′-AATGGGAAACCGACCAGACC-3′
miR-138	F: 5′-GTGTTGTGAATCAGGCCGAC-3′; R: 5′-CGCCTGGAGAGGATGGGTAT-3′
SIRT2	F: 5′-TCAGGATTCAGACTCGGACAC-3′; R: 5′-TGTAGCGTGTCACTCCTTCG-3′
GAPDH	F: 5′-AAAATCACGACTCAGCCCCC-3′; R: 5′-AATGGGAAACCGACCAGACC-3′

## Data Availability

All data generated or analyzed during this study are included within this article. Jinchun Wang and Zhujing Chen are responsible for data reliability.
